# Hyperspectral imaging for quantifying *Magnaporthe oryzae* sporulation on rice genotypes

**DOI:** 10.1186/s13007-024-01215-1

**Published:** 2024-06-08

**Authors:** Angeline Wanjiku Maina, Erich-Christian Oerke

**Affiliations:** Institute for Crop Science and Resource Conservation (INRES) - Plant Pathology, Rheinische Friedrich-Wilhelms University of Bonn, Bonn, Germany

**Keywords:** Blast, Conidia production, *Oryza sativa*, Partial disease resistance, Spectral reflectance

## Abstract

**Background:**

Precise evaluation of fungal conidia production may facilitate studies on resistance mechanisms and plant breeding for disease resistance. In this study, hyperspectral imaging (HSI) was used to quantify the sporulation of *Magnaporthe oryzae* on the leaves of rice cultivars grown under controlled conditions. Three rice genotypes (CO 39, Nipponbare, IR64) differing in susceptibility to blast were inoculated with *M. oryzae* isolates Guy 11 and Li1497. Spectral information (450–850 nm, 140 wavebands) of typical leaf blast symptoms was recorded before and after induction of sporulation of the pathogen.

**Results:**

*M. oryzae* produced more conidia on the highly susceptible genotype than on the moderately susceptible genotype, whereas the resistant genotype resulted in no sporulation. Changes in reflectance spectra recorded before and after induction of sporulation were significantly higher in genotype CO 39 than in Nipponbare. The spectral angle mapper algorithm for supervised classification allowed for the classification of blast symptom subareas and the quantification of lesion areas with *M. oryzae* sporulation. The correlation between the area under the difference spectrum (viz. spectral difference without and with sporulation) and the number of conidia per lesion and the number of conidia per lesion area was positive and count-based differences in rice - *M. oryzae* interaction could be reproduced in the spectral data.

**Conclusions:**

HSI provided a precise and objective method of assessing *M. oryzae* conidia production on infected rice plants, revealing differences that could not be detected visually.

**Supplementary Information:**

The online version contains supplementary material available at 10.1186/s13007-024-01215-1.

## Background

The filamentous ascomycete fungus *Magnaporthe oryzae* B.C. Couch, causal agent of blast disease is of importance to both worldwide rice cultivation and the understanding of host-pathogen interactions [1]. Blast is directly responsible for annual yield losses of up to 10–30%, and complete loss (100%) may occur during an epidemic [2, 3]. Spatial dissemination of the pathogen relies on the production of conidia by multiple cycles of asexual reproduction [4, 5]. As a polycyclic pathogen, multiple disease cycles (8 to 11) from the germination to the production of conidia occur in a growing season [4, 6]. The fungus sporulates continually for about 20 days and a single leaf blast lesion can produce up to 20,000 conidia [1, 7]. Maximum sporulation occurs at an optimum temperature between 25 and 28 ^o^C, relative humidity (RH) above 90%, and extended periods of leaf wetness [8, 9]. The production of *M. oryzae* conidia is a regulated process that requires a period of darkness [1]. The importance of sporulation lies in its contribution to the spread and survival of *M. oryzae*, as the production of conidia is a key step in the development of rice blast epidemics [5, 10]. The cultivation of resistant varieties can decrease the inoculum potential within the field and delays disease epidemics.

Due to the short-lived nature of complete resistance to blast, the development of rice varieties with quantitative resistance (also called partial, rate-reducing, or slow-blasting) is vital for disease resistance breeding [11, 12]. Partial resistance in rice is a form of incomplete resistance characterized by reduced pathogen growth and reproduction [13, 14]. This type of resistance is attributed to different components e.g., reduced infection frequency, longer latent period, lesion size, and reduced sporulation [13–15]. The result is a diminished potential for inoculum production and a decreased likelihood of a blast epidemic. Partial resistance is race non-specific and is controlled by multiple genes, each of which makes a relatively small contribution to the overall resistance, and hence remains effective for a longer time [16, 17]. The small differential interactions between genotypes of rice and *M. oryzae* indicate that partial resistance genes in the host interact on a gene-for-gene basis with genes in the pathogen [18, 19].

Assessing a large number of rice genotypes based on their reaction to all components of partial resistance is difficult because they interact among themselves, and their effects are cumulative during the course of disease development [15]. Moreover, the severity of blast is often measured in the field by the end of the rice growth period and represents the cumulative result of all components of partial resistance [14]. While visual assessment of disease severity is a common method for selecting blast-resistant rice varieties in the field, it may not provide detailed information about the underlying mechanisms of partial blast resistance [14]. It is essential to estimate the contribution of each component of partial resistance to disease development followed by selection for a single component [15, 20]. In the case of partial resistance to leaf blast in rice, the sporulating of *M. oryzae* is an important parameter. Measurement of sporulation in the field is difficult due to the presence of inoculum from adjacent plants. Hence, assessment of sporulation in the greenhouse setting offers the advantage of eliminating any interplot interference, a common phenomenon that often occurs in the field [21].

The traditional microscopic method for counting *M. oryzae* conidia and the use of spore trapping methods in combination with quantitative real-time polymerase chain reaction (qPCR) are labor-intensive, time-consuming, and involve the destruction of leaves [22]. Given the time-dependent nature of conidia production, it is recommended to use an automated method for quantifying fungal sporulation. Various image-based approaches have been proposed for quantifying fungal spores. Qi et al. [23] used micro-images to detect and count the spores of rice blast automatically. Xiaolong et al. [24] automatically counted the urediospores of *Puccinia striiformis* f. sp. *tritici*, the causal agent of wheat stripe rust. Rapid detection of fungal spores in greenhouse crops was accomplished using the complementary metal oxide semiconductor (CMOS) image sensors technique and diffraction fingerprint feature processing [25]. These approaches not only automate the spore detection and counting process but also emphasize the crucial role of precise phenotyping methods in plant disease epidemiology.

Hyperspectral imaging (HSI) has the advantage of rapid and non-destructive detection of plant diseases at the tissue level. It has been used in phenotyping leaf blast of rice [26, 27], the reaction of grapevines to *Plasmopara viticola* [28], *Fusarium* head blight (FHB) of wheat [29], and other plant diseases [30, 31]. Using HSI, Maina and Oerke [27] differentiated blast symptoms into subareas differing in coloration, size, and composition depending on the rice × *M. oryzae* interaction. They observed rapidly enlarging grey tissue localized at the center of the blast symptom in compatible rice × *M. oryzae* interactions. The size of the grey tissue is vital for the assessment of the sporulation rate since sporulation of *M. oryzae* is confined to the central area of blast lesions [32, 33].

HSI captures spectral information in the visible (VIS, 400–700 nm), and near-infrared (NIR, 700–1000 nm) ranges of the spectrum with a high spectral and spatial resolution [34], allowing for the differentiation of subtle changes associated with disease e.g., pathogen sporulation. Both imaging and non-imaging hyperspectral sensors have been used for quantitative analysis of fungal sporulation. Using a non-imaging spectrometer, Ren et al. [35] created a robust yellow rust spore index (YRSI) to detect and quantify wheat yellow rust by closely analyzing how fungal spores influenced the overall leaf spectral response. HSI has been used to detect and diagnose wheat rust disease using reference spectra from spore-scale observations [36]. Oerke et al. [37] successfully used HSI at the microscope scale to quantify the sporulation of *Cercospora beticola* on sugar beet leaves of varying susceptibility. The combination of hyperspectral imaging and computer vision effectively assessed and quantified the sporulation of downy mildew on grapevine leaves [38]. Segmentation of leaf disc images enabled the differentiation of pixels representing downy mildew sporulation from other parts of the leaf. Zhang et al. [39] used a microfluidic chip-based method combined with microscopic HSI to rapidly detect and quantify fungal spores on rice leaves. These studies suggest that HSI is an effective and objective method for assessing fungal sporulation and studying the dynamics of host-pathogen interactions. Nevertheless, most of these investigations relied on the average reflectance spectra from diseased tissues or focused only on a limited number of wavebands to evaluate the sporulation potential of fungal pathogens and the impact of fungal spores on the spectral characteristics of leaves. In breeding for disease resistance, the identification of smaller cultivar differences requires a more precise and reliable method [40]. Incorporating a detailed examination of *M. oryzae* sporulation into the selection process under controlled conditions may help to identify small positive effects related to blast resistance [11, 14, 41]. Accurate estimation of the quantity of inoculum during epidemics demands high precision in data collection and analysis [39, 40]. The characterization and quantification of sporulation using the entire spectrum in the VIS and NIR range may offer insights into the spatial dynamics of pathogen reproduction and disease spread as well as the mechanisms of disease resistance in host plants [37].

In this study, the potential of HSI to measure the sporulation of *M. oryzae*, as a crucial component of the partial resistance of rice to blast was investigated. In greenhouse experiments, the three rice genotypes CO 39, Nipponbare and IR64, varying in the level of susceptibility/resistance to leaf blast, were inoculated with the *M. oryzae* isolates Guy 11 and Li1497. The specific objectives were (a) to determine how host genotypes with different resistance levels affect conidia production of the pathogen; (b) to quantify the sporulation of *M. oryzae* on different rice genotypes by calculating the spectral difference between blast symptoms without and with conidia production, measured as the area under the difference spectrum (AUDS) of the grey tissue; and (c) to establish the relationship between AUDS and the rate of conidia production per lesion and per lesion area, respectively. Eventually, evaluating *M. oryzae* sporulation using HSI would shed light on the complex interactions between rice genotypes and *M. oryzae* isolates. This information can be invaluable in breeding programs to develop rice varieties with durable resistance to blast.

## Materials and methods

### Plant material

Three rice (*Oryza sativa* L.) genotypes differing in susceptibility to *M. oryzae* were used in these experiments; genotype CO 39 (*indica* type*)*, which is highly susceptible to blast was obtained from IRRI, Philippines. Nipponbare (susceptible *japonica* type) and IR64 (resistant *indica* type) were generously provided by Michael Frei, Department of Plant Nutrition, University of Bonn. Seeds of each rice genotype were sown directly in plastic pots (Ø = 9 cm; Kausek, Mittenwalde, Germany) filled with loam soil at 5 seeds per pot. Plants were cultivated under controlled conditions in the greenhouse as described by Maina and Oerke [27].

### Pathogen and inoculation

Two isolates of *M. oryzae* were used separately to inoculate rice leaves in order to take into account the gene-for-gene interactions. Isolate Guy 11 was kindly provided by Didier Tharreau (CIRAD, Montpellier, France), and isolate Li1497 was obtained from BASF SE (Limburgerhof, Germany). The cultivation of *M. oryzae* on rice leaf agar, the production of conidia, and the preparation of inoculum were performed as described earlier [27]. Eighteen days after seeding, rice plants at growth stage (GS) 13 [42] were spray-inoculated with a spore suspension (10^5^ conidia/ml) of *M. oryzae* using a hand sprayer. The inoculated plants were incubated in a dark moist incubation chamber at 25 °C and > 95% RH for 24 h, and subsequently returned to the greenhouse at 60% RH until visible blast symptoms developed. At least two independent experiments were conducted, each with 25 plants per genotype.

### Measurement of conidia production

Seven days post inoculation (d.p.i.), fungal sporulation was induced on mature blast symptoms by incubating diseased rice plants at 100% RH under alternating dark and light conditions (12 h / 12 h) for 2 days. Conidia production was measured at 9 d.p.i. (= 2 days after induction of sporulation). Leaf disks with a single lesion were separately placed into Eppendorf tubes containing tap water (0.5 ml water with 0.01% Tween 20). The tubes were shaken vigorously using a vortex shaker (Vortex-Genie 2, Bohemia, New York, USA) for 10 s to dislodge the conidia from the conidiophores. The number of conidia detached from the lesions was counted under a microscope (magnification 100x) using a Fuchs-Rosenthal chamber (Brand, Wertheim, Germany). Subsequently, the length and width of each lesion were measured to determine its area. Eight lesions per rice genotype × *M. oryzae* interaction were analyzed, and the results were expressed as the number of conidia per lesion and the number of conidia per lesion area [mm^2^].

### Microscopy

To study the characteristics of *M. oryzae*, a Leica Leitz DMR stereomicroscope (Leica Microsystems, Wetzlar, Germany) was used for light microscopy. Leaf sections with a single blast lesion were used to visualize the conidiophores and conidia on the surface of the sporulating lesions. Images were recorded using the software Discus (Technisches Büro Hilgers, Königswinter, Germany). For staining for fluorescence microscopy, leaf sections with a single blast lesion were cut and placed in 2 ml Eppendorf tubes containing 1 ml 10% KOH. About 50 µL of Silwet® L-77 was added and the Eppendorf tubes were wrapped in aluminum foil for approximately 90 min. The leaf samples were placed onto clean glass slides, then a drop of calcofluor white was added, covered with a coverslip, and left to absorb the stain for 1 min. Samples were observed under a Leica SP8 confocal laser scanning microscope (Leica Microsystems, Wetzlar, Germany).

### Hyperspectral measurements and image analysis

To measure spectral differences between blast lesions without and with conidia, hyperspectral images were recorded using a spectral line scanner (spectral camera PFD V10E, Spectral Imaging Ltd., Oulu, Finland) as previously described [27]. Images of fully developed blast lesions on leaves of rice genotypes were recorded 7 days post inoculation (d.p.i.) as well as 2 days after the induction of sporulation (= 9 d.p.i.). Eight lesions per rice genotype × *M. oryzae* interaction were analyzed for each time of image recording (before and after sporulation). The reflectance of hyperspectral images was calculated by normalizing the images relative to the reflection of a 100% white reference standard (Zenith Polymer Target, SphereOptics GmbH, Uhldingen, Germany) and to a dark current measurement using ENVI 5.3 + IDL 8.3 (ITT Visual Information Solutions, Boulder, CO). Because of noise at the extremes of the spectra, only wavelengths between 450 and 850 nm (140 wavebands) were used for spectral analysis and the resultant spectral signals were smoothed using a Savitzky-Golay filter [43].

All tissue types (= endmember classes) of the infected rice leaves - healthy (green) tissue, dark brown tissue, grey tissue, chlorotic tissue, grey green tissue, and light brown tissue - were characterized spectrally for images recorded before (7 d.p.i.) and 2 days after sporulation (= 9 d.p.i.). For each tissue type of the rice × *M. oryzae* interactions, at least 4 different areas were used per image to extract the mean (= typical) spectrum, which was stored in a spectral library, the collection of reference spectra (= endmember collection for the spectral angle mapper (SAM) algorithm). The number of pixels per region of interest (ROI) ranged from 2,600 to > 100,000 for healthy (green) tissue and from 50 to > 5,000 pixels for blast symptom subareas. Spectral signatures of different blast symptom subareas (= endmember classes) from eight representative lesions per rice genotype × *M. oryzae* interaction were extracted separately for each time of image recording. The spectral endmembers of the spectral libraries were used for the supervised classification of pixels by using the SAM algorithm [44].

### Spectral quantification of *M. oryzae* sporulation

**Fig. 1:**
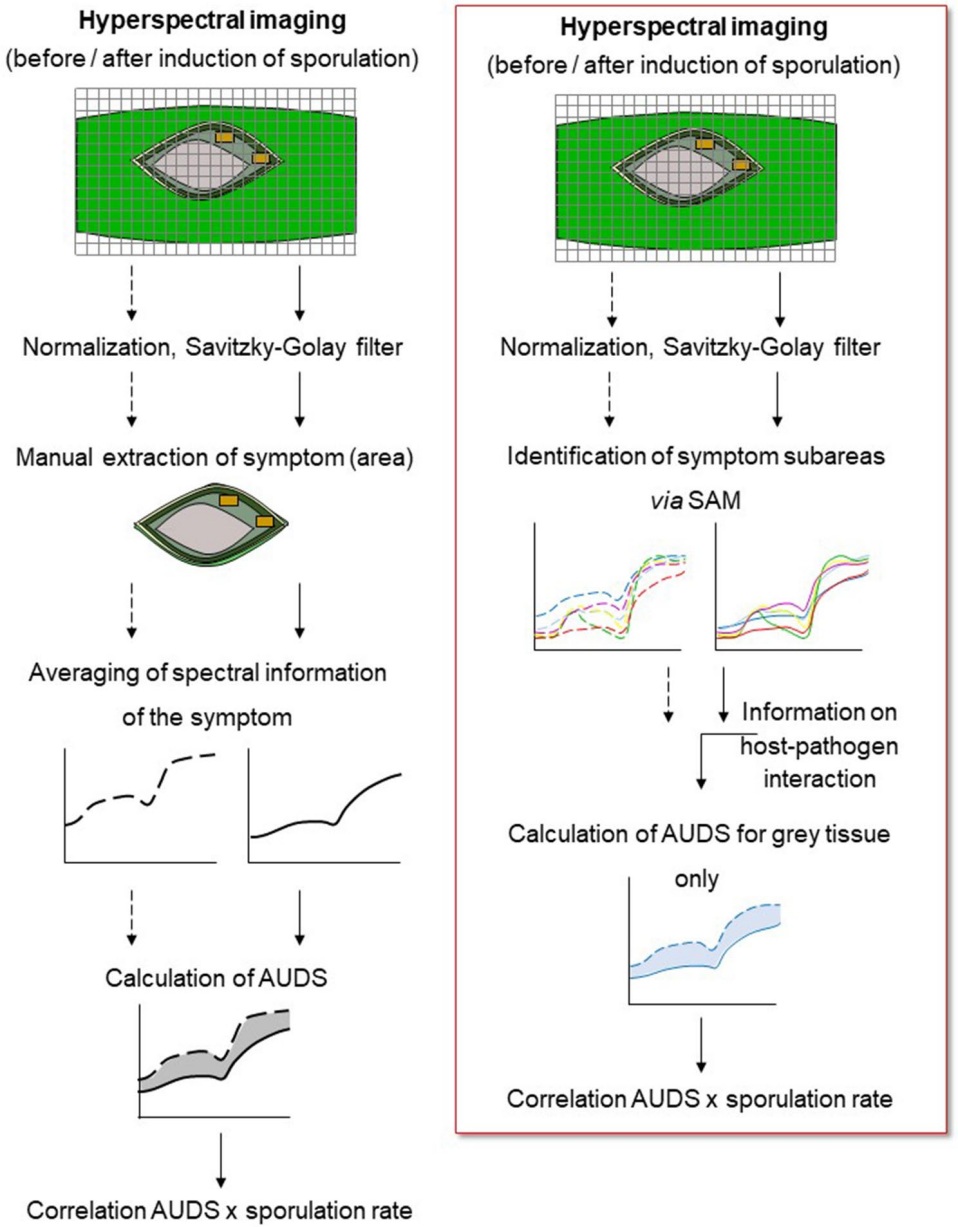
Workflow for the quantification of *M. oryzae* sporulation using hyperspectral imaging data. In contrast to the workflow for assessing fungal sporulation by characterizing changes in the mean spectrum per disease symptom defined manually (left), the spectral angle mapper (SAM) was applied onto images after normalization and spectral smoothing by the Savitzky-Golay filter (right). Based on information from literature, the spectral difference (AUDS) between images recorded before and after the induction of *M. oryzae* conidia production was calculated only for the central grey tissue of blast symptoms

For the spectral quantification of sporulation, reflectance spectra of grey tissue without (7 d.p.i.) and with sporulation (9 d.p.i.) were extracted from the same eight representative lesions from rice genotypes CO 39 and Nipponbare (workflow see Fig. 1).

The spectral difference between grey tissue without and with sporulation, respectively, was quantified as the area under the difference spectrum (AUDS) (= area between the spectra of grey tissue recorded 7 and 9 d.p.i., respectively). The resulting AUDS [%/100.nm], characterizes the spectral modification caused by *M. oryzae* sporulation across the visible (VIS) and near-infrared (NIR) ranges of the spectrum.

### Statistical analysis

Statistical analyses were conducted using the R software [45]. A standard analysis of variance (ANOVA) was performed to determine the significance of differences in the number of conidia per lesion, number of conidia per lesion area, number of pixels of grey tissue, and in the area under the difference spectrum. Mean separation was performed using Tukey’s honest significant difference test (*P* = 0.05). Correlations between AUDS and the number of conidia per lesion and number of conidia per lesion area were calculated using Pearson’s correlation coefficient with the significance threshold set at *P* ≤ 0.05. The number of replicates (n) used for statistical analysis is given in the results section.

## Results

### Effect of rice genotypes on the intensity of *M. oryzae* sporulation

**Fig. 2:**
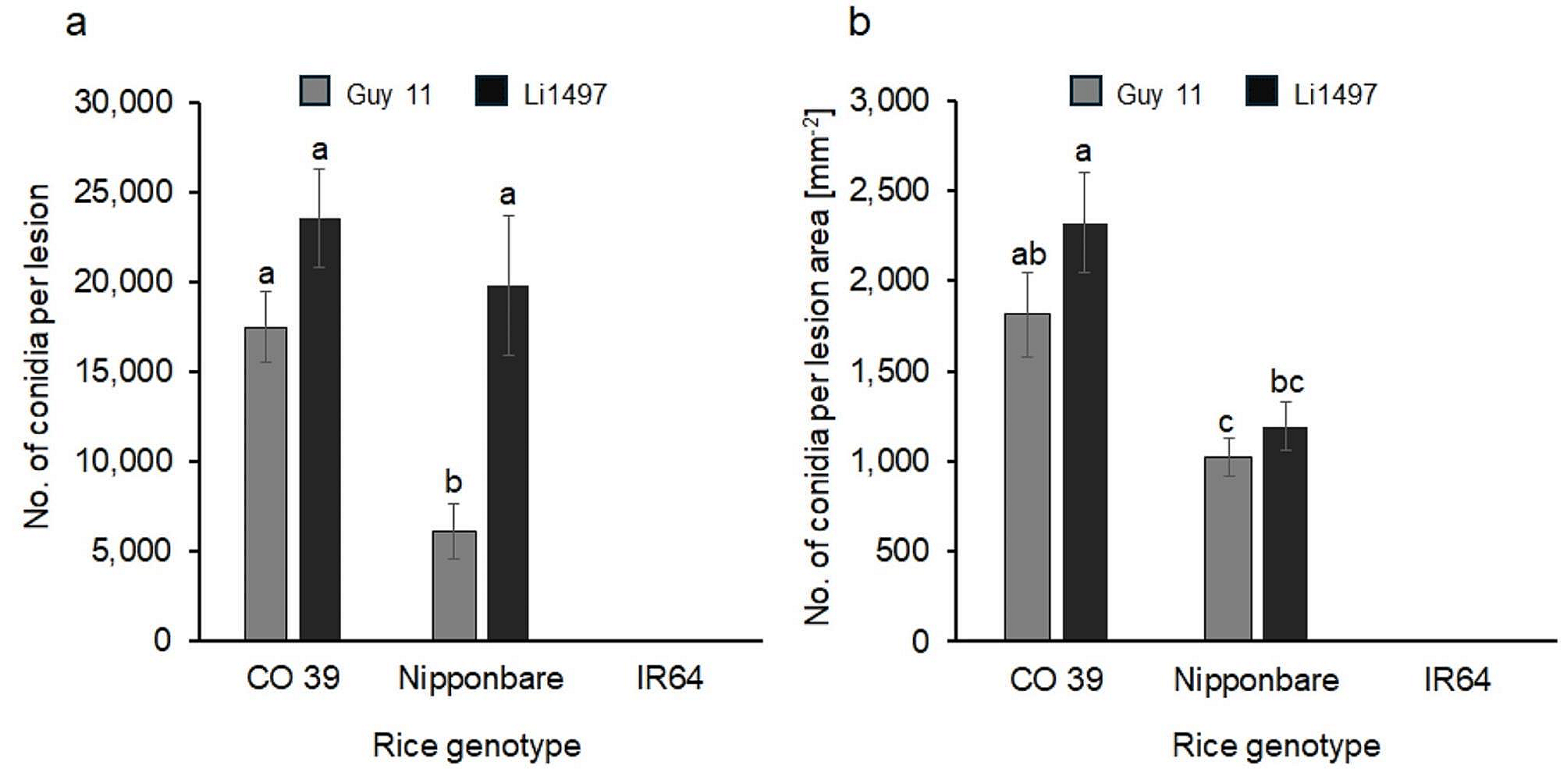
Conidia production of *M. oryzae* isolates Guy 11 and Li1497 on the three rice genotypes CO 39, Nipponbare, and IR64 differing in susceptibility to leaf blast; (**a**) number of conidia produced per lesion; (**b**) number of conidia produced per lesion area. Sporulation was assessed 9 days post inoculation (= after 2 days of induction of conidia production). Columns with the same letter were not significantly different (*P* = 0.05, Tukey’s honestly significant difference test). Bars represent standard error of the mean (*n* = 8)

The production of conidia of *M. oryzae* isolates Guy 11 and Li1497 on rice genotypes CO 39, Nipponbare, and IR64 was quantified microscopically (Fig. 2). The number of conidia produced per lesion was counted 2 days after diseased rice plants had been incubated at 100% RH (= 9 d.p.i.). On rice genotypes CO 39 and Nipponbare, more than 2,000 conidia per lesion were produced within 2 days. The number of conidia per lesion differed among rice genotype × *M. oryzae* interactions. It was significantly (*P* < 0.05) higher in highly susceptible genotype CO 39 infected by isolates Guy 11 and Li1497 and genotype Nipponbare susceptible to isolate Li1497 compared to genotype Nipponbare which was only moderately susceptible to *M. oryzae* isolate Guy 11. When sporulation was expressed as conidia per lesion area, *M. oryzae* produced more conidia on genotype CO 39 than on Nipponbare for all interactions. Statistically significant (*P* < 0.05) differences were observed between CO 39 infected with Li1497 and Nipponbare infected with Guy 11. Both *M. oryzae* isolates did not produce conidia on the resistant genotype IR64 (Fig. 2).

### Morphological characteristics of sporulating lesions, conidiophores, and conidia

**Fig. 3:**
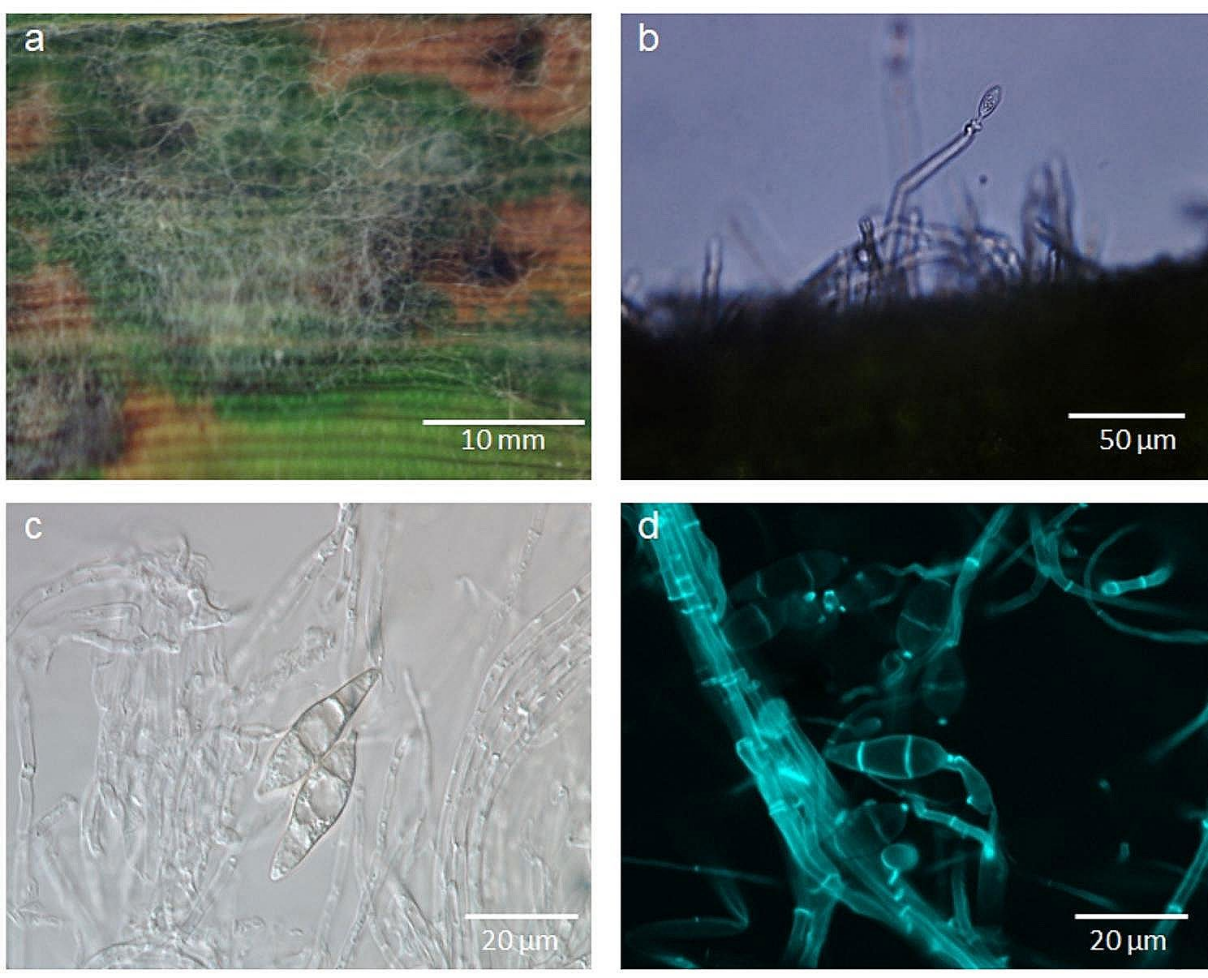
Characteristics of *M. oryzae* on the highly susceptible rice genotype CO 39, 9 days post inoculation; (**a**) typical blast lesion with grey aerial mycelium; (**b**) hyaline conidiophore with conidia protruding from the surface of blast lesion, (**c**) morphology of *M. oryzae* conidia in bright field microscopy, (**d**) image of conidiophores and conidia from confocal laser scanning microscopy (staining with calcofluor)

The characteristics of typical sporulating blast lesions on susceptible rice genotypes were assessed 9 d.p.i. (Fig. 3).

Incubation of rice plants for two days under high RH led to the formation of aerial mycelia of *M. oryzae* that spread on the surface of the lesion (Fig. 3a). Microscopic examination revealed the presence of the conidiophores (bearing conidia) protruding from the lesion surface. The conidiophores were elongated, slender, and septate (Fig. 3b). The conidia of *M. oryzae* were hyaline (translucent), pyriform or pear-shaped with a distinct narrow apex (pointed end) and a rounded base, and had two septa, three cells (Fig. 3c and d).

### Phenotypes of typical blast symptom types during conidia production

**Fig. 4:**
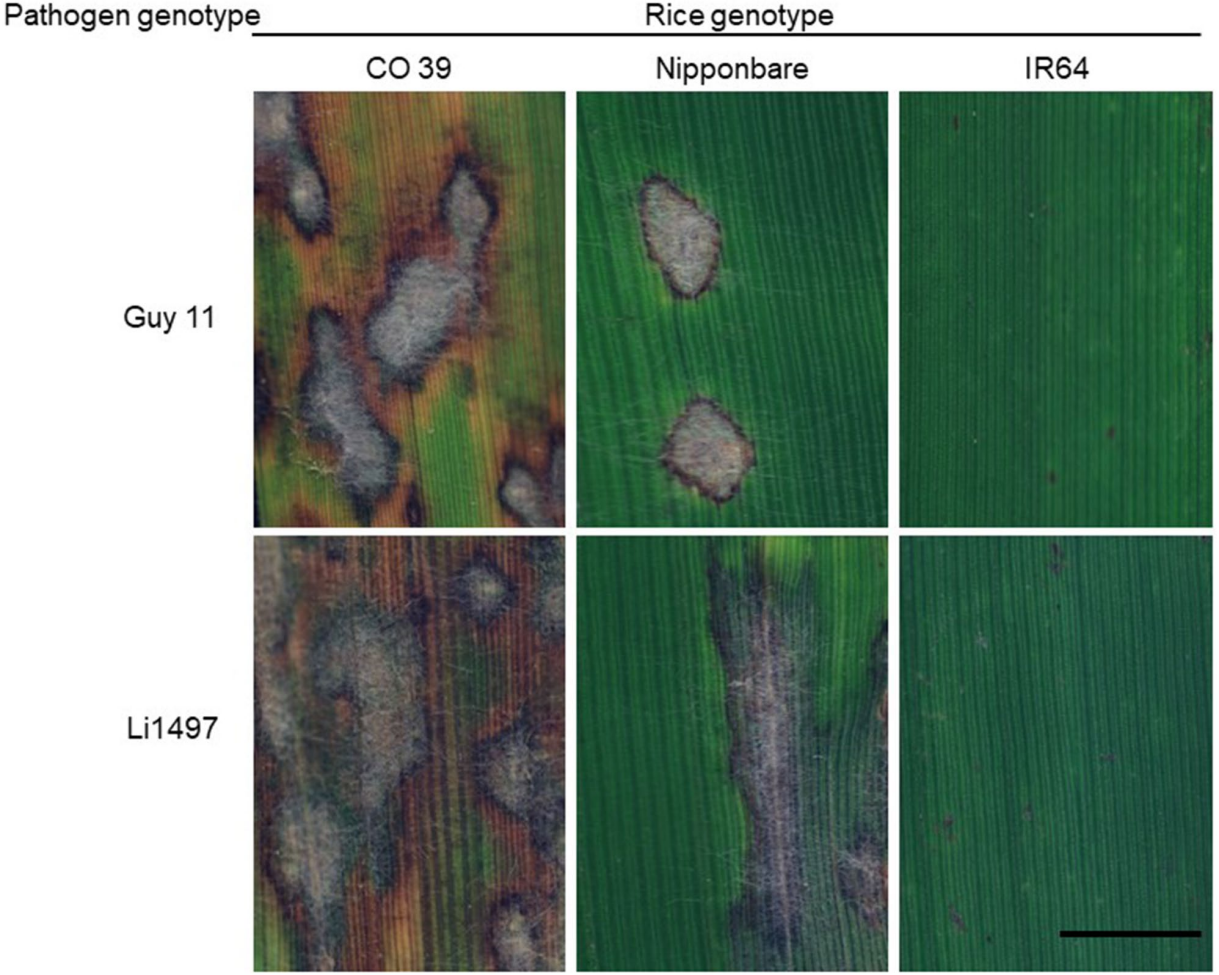
Phenotypes of leaf blast symptoms on rice genotypes CO 39, Nipponbare, and IR64 infected with *M.oryzae* isolates Guy 11, and Li1497, respectively. Images were recorded after incubation of rice plants under 100% RH for 2 days to induce sporulation, corresponding to 9 days post inoculation (bar size = 10 mm)

During conidia production, the typical blast symptoms exhibited variation depending on the specific interaction between the genotypes of host and pathogen and the size, shape, and color of blast symptom subareas varied within leaves (Fig. 4). The type of blast symptom and the lesion area had an influence on the production of conidia by *M. oryzae*. In case of the resistant rice genotype IR64, the blast lesions on the leaf surface were small dark brown spots. There was no formation of a sporulating lesion center on resistant genotype IR64, and consequently, no subsequent production of conidia.

In contrast, leaves of CO 39 - highly susceptible to isolates Guy 11 and Li1497 - and Nipponbare - susceptible to Li1497, but only moderately susceptible to Guy 11 - displayed different patterns of blast symptoms. In highly compatible interactions, the characteristic blast lesions were larger, elliptical, or spindle-shaped and rapidly expanded due to vigorous *M. oryzae* growth during the two days of incubation for sporulation. The blast symptoms on the moderately resistant genotype were relatively smaller than in the compatible and highly compatible interactions. The center of blast lesions was characterized by distinct greyish tissue, surrounded by grey green tissue with a brown margin and chlorotic tissues and by light brown tissue. The grey tissue in the center of lesions could be attributed to the growth of superficial mycelia, the formation of conidiophores, and the production of conidia (Fig. 4).

### Spectral assessment of blast symptom subareas

**Fig. 5:**
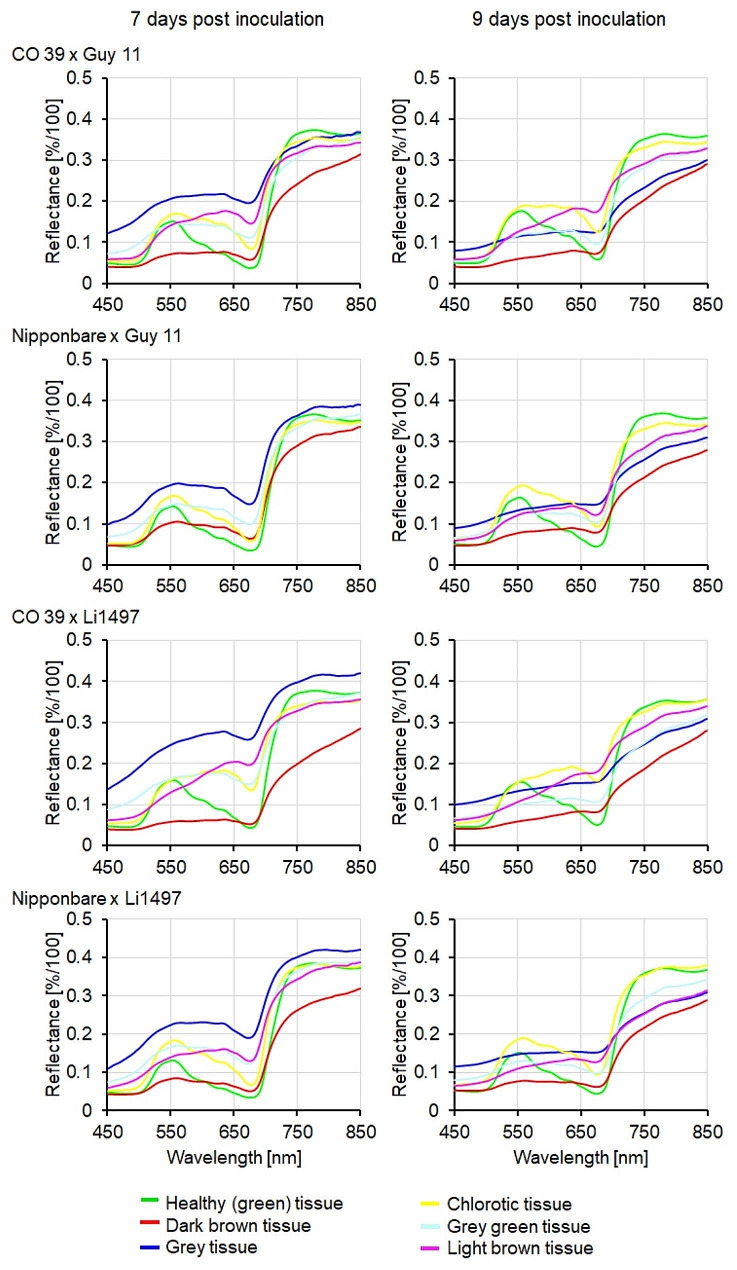
Reference spectra of healthy (green) tissue and of blast symptom subareas from leaves of rice genotypes CO 39 and Nipponbare infected with *M. oryzae* isolates Guy 11 and Li1497, respectively. Spectra of healthy tissue and of blast symptom subareas were extracted before (7 d.p.i., left) and 2 days after induction of sporulation (= 9 d.p.i., right)

The average reflectance spectra in the VIS and NIR spectral range of healthy (green) tissue and specific subareas of the blast symptoms were manually extracted before sporulation (7 d.p.i.) and 2 days after induction of sporulation (= 9 d.p.i.) (Fig. 5, Additional file 1: Fig. A1). The differences in the spectral signatures were used for the classification of lesion pixels. Although similar, the spectra of healthy (green) tissue significantly differed between rice genotypes in the VIS and NIR ranges (Additional file 2: Fig. A2). The spectral signatures of different blast symptom subareas considerably differed from healthy (green) tissue as well as from each other. The generation of reference spectra, therefore, required the definition of regions of interest (ROIs) for each blast symptom subarea for the individual rice genotypes (Fig. 5).

**Fig. 6:**
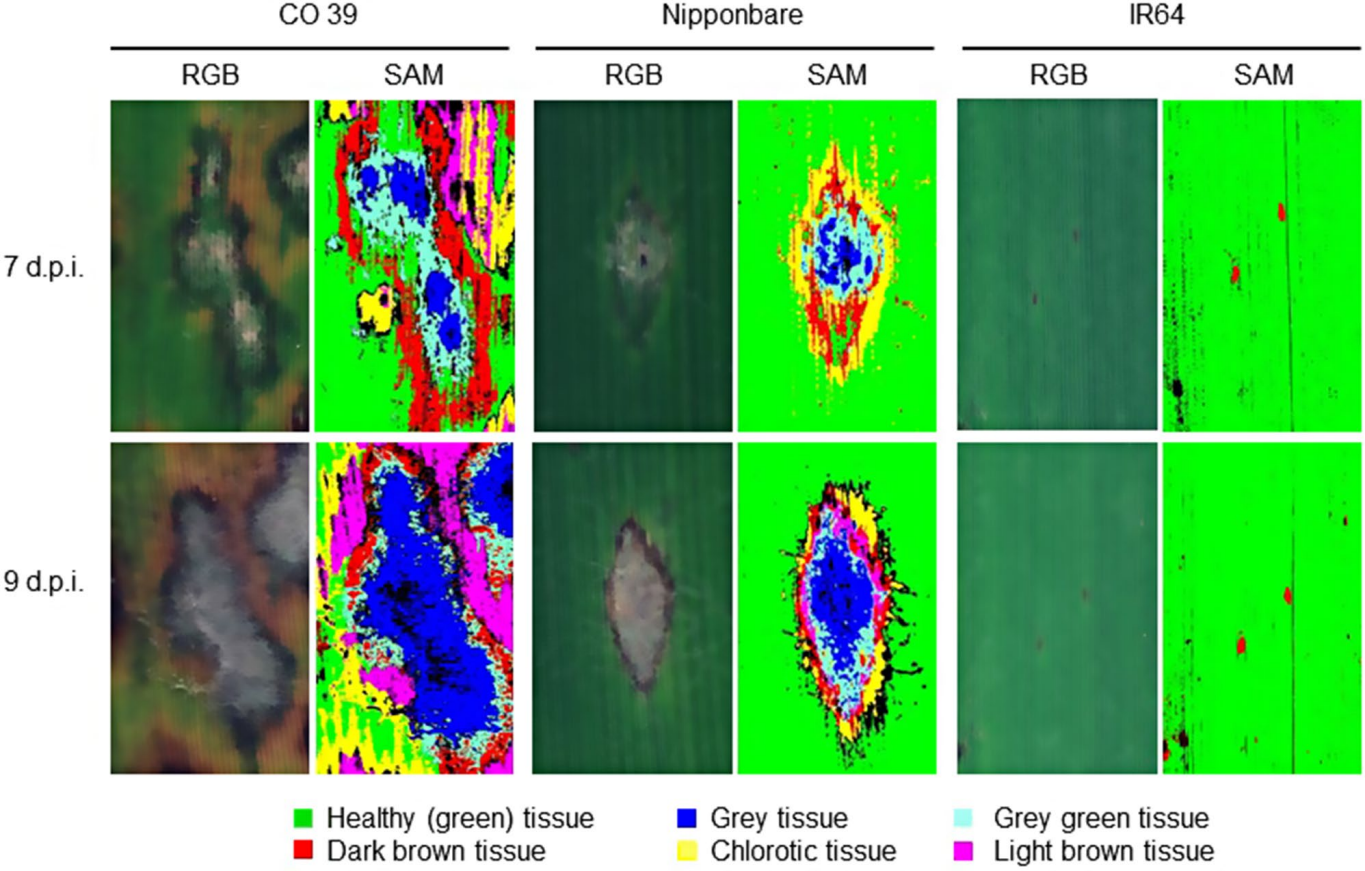
Sporulation of *M. oryzae* isolate Guy 11 on leaves of the rice genotypes CO 39, Nipponbare, and IR64 as quantified by hyperspectral imaging. Images illustrate RGB picture and pseudo-color results of spectral angle mapper (SAM) classification of blast lesions before induction of sporulation (7 days post inoculation) and with sporulation (9 d.p.i.) induced by incubation of rice plants under 100% RH for 2 days. Different subareas of blast symptom types were classified for each rice genotype × *M. oryzae* interaction

**Fig. 7:**
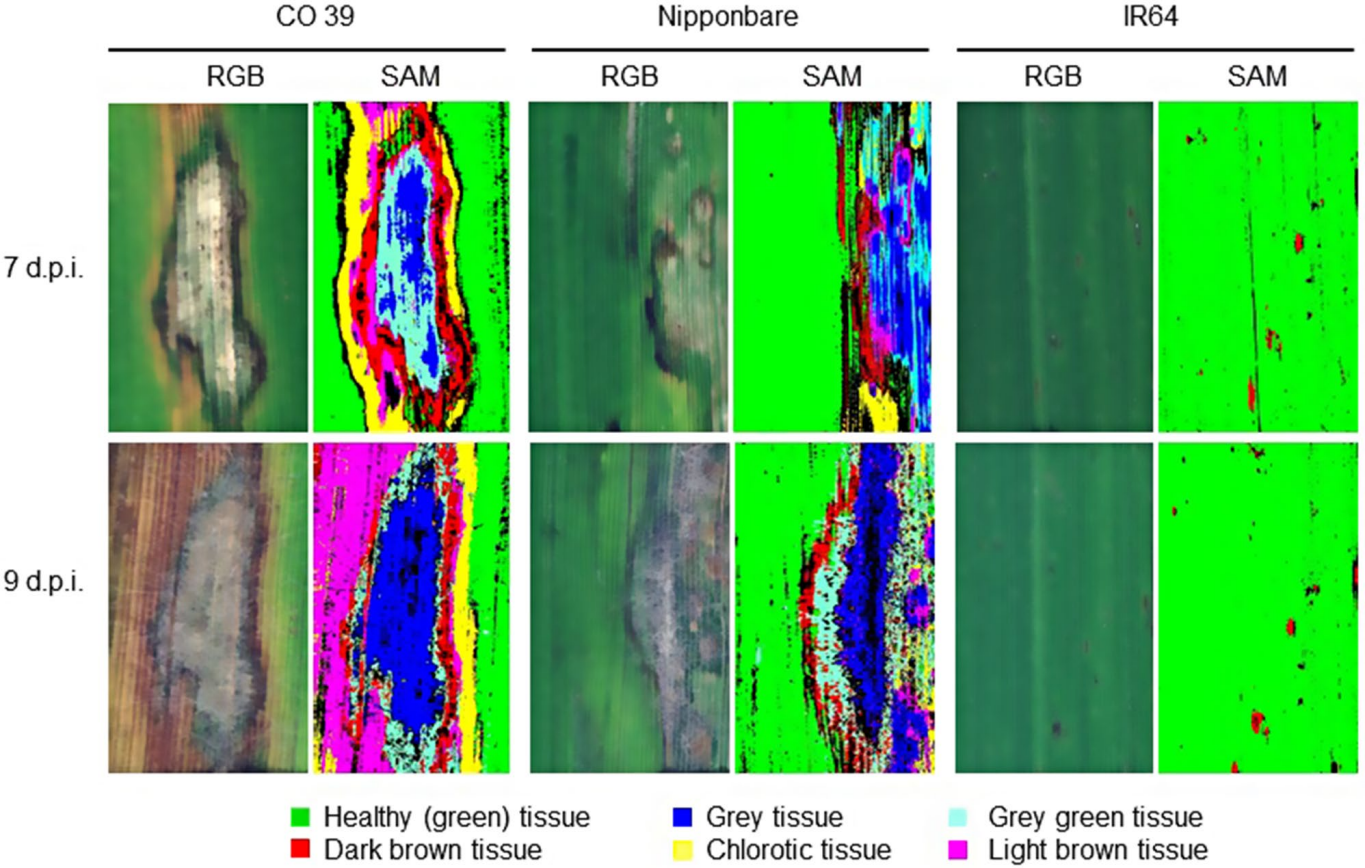
Sporulation of *M. oryzae* isolate Li1497 on leaves of the rice genotypes CO 39, Nipponbare, and IR64 as quantified by hyperspectral imaging. Images illustrate RGB picture and pseudo-color results of spectral angle mapper (SAM) classification of blast lesions before induction of sporulation (7 days post inoculation) and with sporulation (9 d.p.i.) induced by incubation of rice plants for 2 days under 100% RH. Different subareas of blast symptom types were classified for each rice genotype × *M. oryzae* interaction

Using the specific spectra of green tissue and blast symptom subareas as reference spectra (endmembers), the supervised classification of images taken before and after induction of *M. oryzae* sporulation was done using the SAM algorithm (Figs. 6 and 7). As visualized by the pseudo-color images, SAM was able to differentiate blast symptoms into different subareas based on the color (and structure) of the tissue and depending on the rice × *M. oryzae* interaction.

In genotype IR64 resistant to both *M. oryzae* isolates used in this study, only small dark brown spots were classified without differentiation into subareas. For the interaction between CO 39 × Guy 11 or Li1497, Nipponbare × Li1497, and Nipponbare × Guy 11, blast symptoms had a typical zonation into different subareas that included dark brown tissue, grey tissue, grey green tissue, chlorotic tissue, and light brown tissue, indicating the complex nature of the interaction between host and pathogen. Each blast symptom subarea had specific spectral signatures as shown in Fig. 5 and Additional file 1: Fig. A1. Spectral reflectance of the grey tissue was significantly reduced within the two-day incubation period. In contrast, no significant differences were observed in the spectra of the other blast symptom subareas (dark brown, chlorotic, grey green, and light brown tissues) and healthy tissue during the incubation period, suggesting a specific response in the grey tissue. Grey tissue was confined to the central area of blast lesions for both CO 39 and Nipponbare genotypes, irrespective of the infecting *M. oryzae* isolate. The repeated measurements on the same blast symptom type 7 and 9 d.p.i. indicated an increase in lesion size and a change from one symptom type (= spectral class) to another e.g., green tissue to chlorotic tissue, grey green tissue to grey tissue, chlorotic tissue to light brown tissue due to fungal growth (colonization), growth of surface mycelial on the lesion, formation of conidiophores, and production of conidia (Figs. 6 and 7). The strongest changes in coloration were observed in highly compatible interactions.

### Effects of *M. oryzae* sporulation on reflectance spectra of blast lesions

**Fig. 8:**
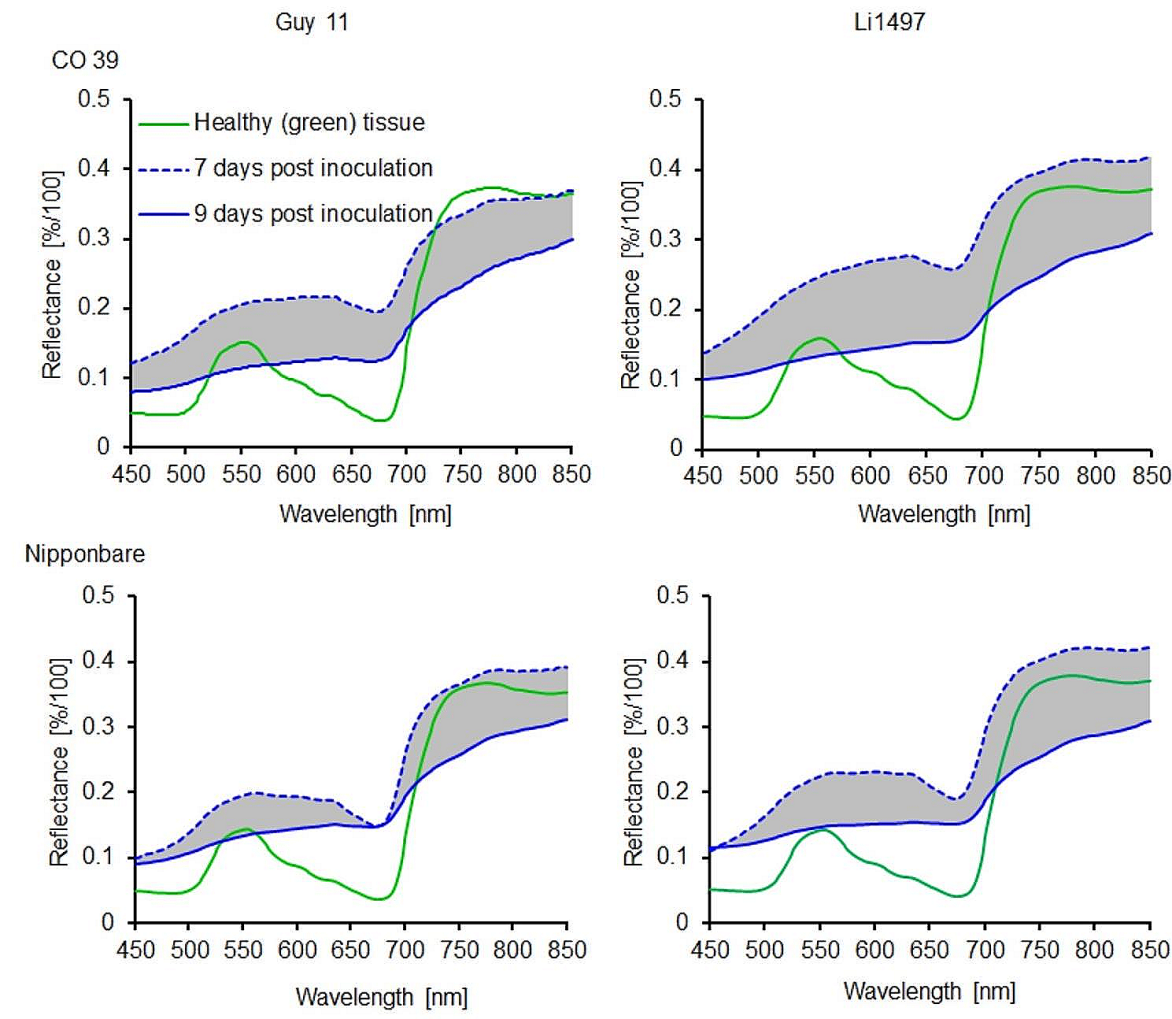
Spectral signatures of healthy (green) tissue and grey tissue (sporulating area) of rice genotypes CO 39 and Nipponbare infected with *M. oryzae* isolates Guy 11 and Li1497, respectively. The spectral signatures were extracted before (7 days post inoculation) and with sporulation (9 d.p.i.), (*n* = 8). The shaded area represents the area under difference spectrum for the grey tissue

**Fig. 9:**
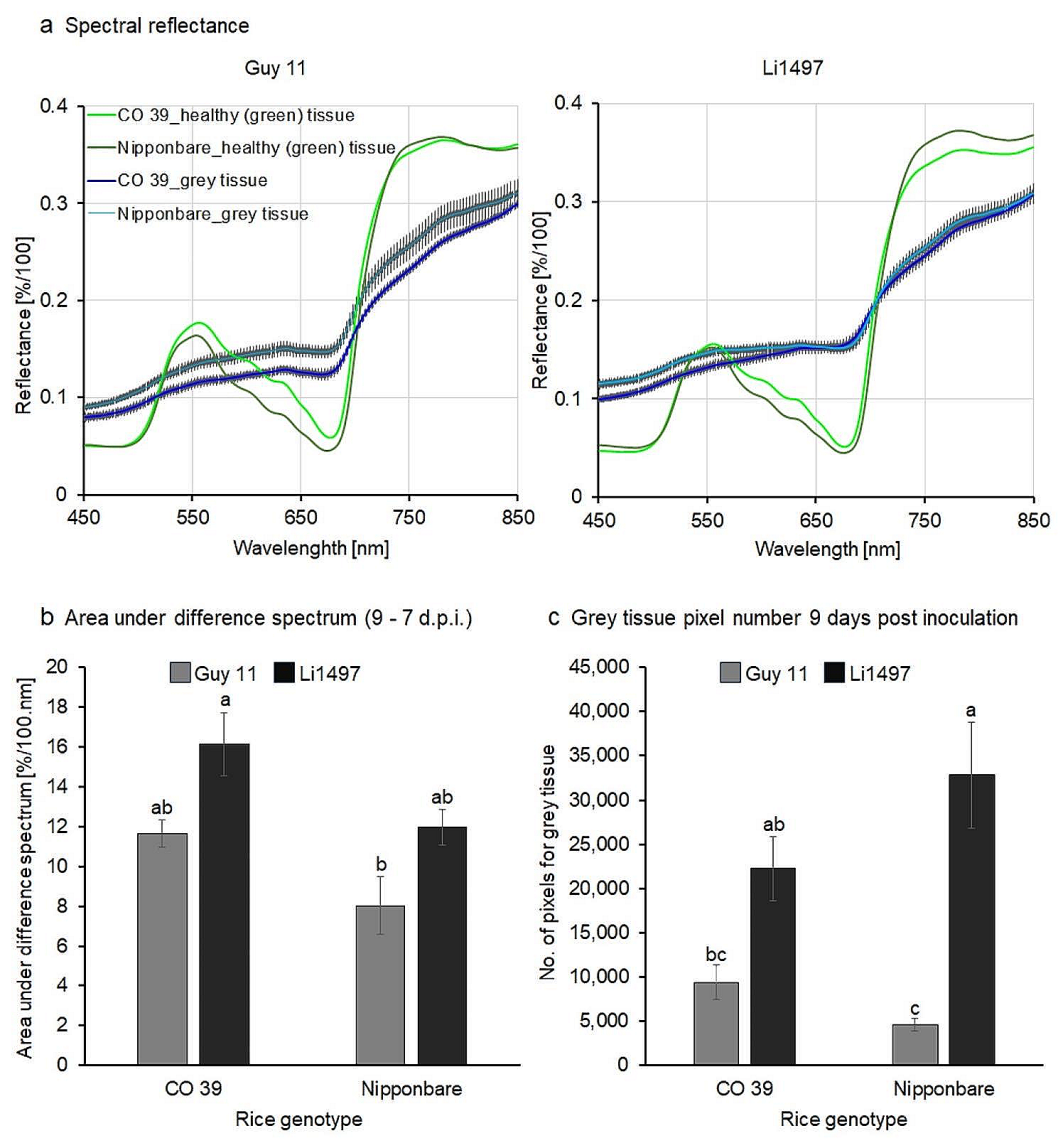
(**a**) Comparison of spectra for healthy (green) tissue and grey tissue (= sporulating area) from rice genotypes CO 39 and Nipponbare infected with *M. oryzae* isolates Guy 11 and Li1497, respectively, 9 days post sporulation. Bars represent standard error of the mean for each waveband (*n* = 8); (**b**) area under the difference spectrum calculated from spectra taken before (7 d.p.i.) and with sporulation (9 d.p.i.), respectively; (**c**) Quantitative assessment of the pixels representing grey tissue (= sporulating area) from CO 39 and Nipponbare, 9 days post sporulation. Values with the same letter are not significantly different (Tukey’s honestly significant difference test (*p* = 0.05, *n* = 8 [spectra]). Bars indicate standard error of the mean

The average reflectance spectra of grey tissue from blast infected leaves of rice genotypes CO 39 and Nipponbare were extracted before the induction of sporulation and compared to the reflectance extracted 2 days after induction of sporulation (Fig. 8). Compared to the green tissue, the spectral signature of grey tissue was characterized by higher reflectance across the visible (450–700 nm) and near-infrared (700–850 nm) range. After incubating infected rice plants under 100% RH for the induction of sporulation, the formation of aerial mycelia, conidiophores, and conidia by *M. oryzae* on the surface of the grey tissue led to a strong decrease in reflectance across the full spectral range. Changes in spectra before and after induction of sporulation were significantly higher for rice genotype CO 39 than for Nipponbare, irrespective of the *M. oryzae* isolate involved (Fig. 8) indicating a genotype-specific difference in the spectral characteristics (Figs. 8 and 9a).

The mean value of the area under difference spectrum was highest for the interaction CO 39 × Li1497 and was significantly (*P* < 0.05) lower for genotype Nipponbare infected by Guy 11 (Fig. 9b). The area of grey tissue (i.e., sporulation area) 9 d.p.i., as classified by SAM algorithm ranged from 2,526 to 59,819 pixels, revealing significant differences (*P* < 0.05) between rice genotypes (Fig. 9c).

**Fig. 10:**
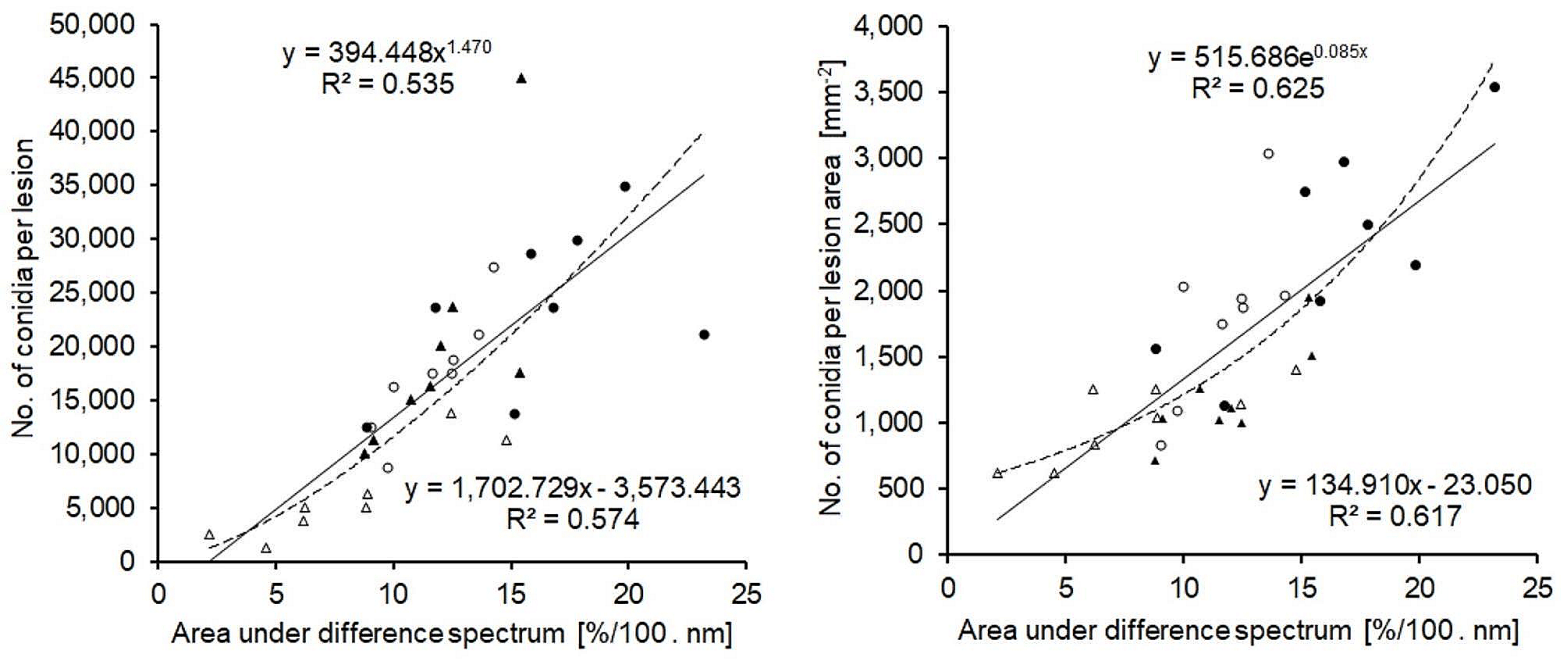
Relationship between the area under difference spectrum (AUDS) and the number of *M. oryzae* conidia produced on lesions of two rice genotypes differing in susceptibility to blast. Correlation between AUDS from spectra recorded without (7 d.p.i.) and with (9 d.p.i.) sporulation, respectively, and the counted number of conidia per lesion (left) and the number of conidia per lesion area (right) (*n* = 32). Linear (solid line) and non-linear relationships (dashed line; power function and exponential function, respectively) with the highest coefficients of determination

The relationship between these spectral differences and the actual *M. oryzae* conidia production was assessed using Pearson correlation analysis. The AUDS values showed a significant, positive correlation to the number of conidia produced per blast lesion and the number of conidia produced per lesion area (Fig. 10). For the number of conidia per lesion, the linear relationship (R² = 0.574; *r* = 0.758 significantly different from 0, *P* < 0.05) was better than the power function (R² = 0.535); for the number of conidia per lesion area, the exponential function (R² = 0.625) was marginally better than the linear function (R² = 0.617; *r* = 0.785 significantly different from 0, *P* < 0.05) in describing the relationship to AUDS.

When the four host-pathogen interactions were analysed separately, the coefficient of determination for the correlation between AUDS and sporulation per lesion area differed considerably and the slope of the regression lines - range 58x to 257x - indicated to differences in compatibility; cv. Nipponbare limited *M. oryzae* sporulation stronger than CO 39, the sporulation rate of isolate Li1497 was higher than that of Guy 11 (Additional file 3: Fig. A3).

## Discussion

This study investigated the potential of HSI to measure the spore production of *M. oryzae* on rice genotypes differing in susceptibility to the blast pathogen. The rate of conidia production per lesion and conidia production per lesion area differed depending on the compatibility between rice genotypes and *M. oryzae* isolates. Resistant genotype IR64 restricted fungal colonization to tiny brown spots and no sporulation was detected, while highly compatible host-pathogen interactions resulted in significantly higher conidia densities than moderately compatible interactions. Significant differences in conidia density of *M. oryzae* on cultivars of varying susceptibility to blast have been reported in rice [10], wheat [46] as well as in other host-pathogen interactions such as the sugar beet − *Cercospora beticola* [37, 47]. Significant differences in the pathogen’s ability to produce conidia on rice genotypes are attributed to the genetic constitution of cultivars [10] and to the interaction between host-pathogen genotypes [41]. Rice genotype Nipponbare infected by *M. oryzae* isolate Guy 11 possessed stable slow-blasting attributes as exhibited by reduced sporulation rate. Thus, by identifying rice genotypes that result in lower fungal sporulation under controlled conditions, breeders can select traits of quantitative disease-resistance and develop improved rice varieties with enhanced field - resistance to rice blast.

Sporulation of *M. oryzae* generally occurs under environmental conditions characterized by high RH and leaf wetness [33, 48]. Under such favorable conditions, the pathogen is able to produce an abundance of conidia on rice leaves, reaching hundreds of thousands [1, 6]. Thus, incubation of susceptible rice plants with typical blast lesions under 100% RH for 2 days combined with alternating periods of darkness and light, lead to the formation of massive mycelium on the surface of leaf blast lesions and the subsequent production of conidiophores with conidia. The mycelium, conidiophores, and conidia collectively contributed to the characteristic grey appearance of sporulating lesions with conidia production typically confined to the central region of blast symptoms as reported in previous studies [49, 50]. The size of this subarea varied among symptoms of a leaf and among rice genotypes [27]. These results suggest that conidia production is spatially restricted and can be influenced by both the size of the grey tissue in the center of leaf blast lesion and the genetic characteristics of the rice plant [32]. Similar observations on *Cercospora beticola* infection of sugar beet have been reported [37].

Mycelial growth of *M. oryzae* is a process essential for both the size of sporulating tissue and the production of conidia, subsequently influencing the patterns and dynamics of disease epidemics [51]. Slow-blasting components such as smaller sporulating lesion areas and low sporulation rate were observed in the interaction between rice genotype Nipponbare and *M. oryzae* isolate Guy 11 as compared to highly compatible interactions, revealing a gene-for-gene specific manner in which minor genes in host genotypes and pathogen isolates operate [19]. Thus, variability in the host-pathogen interactions and genetic diversity among rice plants and pathogen isolates influence the relationship between lesion size and spore production [41, 51]. A substantial increase in blast lesion size on rice leaves within two days of incubation was consistent with observations on *Cercospora* leaf spot on sugar beet [37]. This phenomenon highlights the active mechanisms during fungal colonization, in which the interaction between pathogen and host tissue plays a pivotal role in promoting sporulation. The production of conidia by *M. oryzae* relies on nutrient availability. *M. oryzae* actively exploits the host’s resources to fuel its sporulation process, leading to a more extensive and intensified production of conidia [52].

The spectral characterization of green tissue and blast symptom types in this study demonstrated that reflectance spectra of symptom subareas differed from each other in definite regions of the electromagnetic spectrum. Green tissue displayed typical low reflectance in the VIS, a sharp reflectance increase in the red edge inflection point, and a high reflectance plateau in the NIR [27]. Reflectance spectra of healthy leaf tissue of rice genotype CO 39 differed from those of Nipponbare in the VIS and NIR range. The differences in the VIS range may be attributed to differences in pigmentation, e.g., Nipponbare leaves were the greenest and exhibited low reflectance, in contrast to CO 39, which had higher reflectance. Additionally, differences in tissue structure influenced NIR reflectance especially in rice genotypes infected by *M. oryzae* isolate Li1497. The broad and thin leaves of CO 39 were more affected by pathogen colonization than the narrow and thicker leaves of Nipponbare resulting in a lower NIR reflectance. Different subareas of blast symptoms were associated with changes in reflectance across the full range of the spectrum. The tissue in the center of blast symptoms on rice genotypes CO 39 and Nipponbare at 7 d.p.i. was characterized by an increase in the reflectance over the full range of the spectrum and confirmed an earlier report [27]. On the other hand, the formation of superficial mycelia with conidiophores and conidia on the grey leaf tissue resulted in a reduction of spectral reflectance of this symptom subarea 2 days after the induction of conidiation.

Using separate reference spectra for each image enabled the differentiation of reflectance spectra of the grey tissue before and after sporulation, respectively, across the full spectrum, highlighting distinct spectral characteristics associated with sporulation. Conidia of *M. oryzae* typically develop on conidiophores which usually emerge through stomata but can also breach or directly erupt through the host cuticle from underlying cells of the pathogen [53]. Surface mycelium, conidiophores, and conidia are hyaline and make only a small contribution to the overall spectral reflectance of sporulating lesions. The decreased reflectance of the sporulating leaf area indicates a substantial reduction in the reflectance of the underlying rice tissue due to necrosis and / or tissue damage. The decrease in reflectance is an observable marker for the impact of the pathogen on the host tissue, reflecting the physiological alterations and damage induced during *M. oryzae* colonization of host tissue. In *Cercospora* leaf spot of sugar beet, the sporulation of *C. beticola* reduced the reflectance of lesions over the full range of the spectrum because of the formation of dark-pigmented pseudo stromata and conidiophores [37].

Examining symptom phenotypes may provide valuable insights into the underlying physiology of host-pathogen interactions [54]. The results of SAM classification indicated that the resistant rice genotype IR64 was characterized by small dark brown infection sites without differentiation into distinct zones and colors. The categorization of blast symptoms into different subareas revealed lesions with larger grey centers in highly compatible interactions than in less compatible interactions. Changes in blast symptom composition during the incubation under 100% RH for sporulation resulted from an increase in the size of lesion subareas and a shift from one symptom type to the other. Blast symptoms differ in their spatial and spectral characteristics and the impact of fungal growth and colonization of the leaf tissue during pathogenesis on spectral signatures was correlated with the rate of symptom expansion [27] and as demonstrated here - the production of conidia. However, the spectral differences among rice genotypes made it necessary to use genotype-specific reference spectra of symptom subareas thus limiting the use of general reference spectra (endmembers) in the supervised classification of sporulation structures.

The sporulation of blast lesions is a function of the size of grey tissue in the center of lesions [49] and the limited size of this center is associated with partial resistance to leaf blast [19]. In the present study, the average size of this grey tissue identified and quantified by SAM classification varied between rice genotypes and allowed the assessment of the sporulation area. The area under difference spectrum is a quantitative indicator of the changes in reflectance due to fungal sporulation and characterized genotype CO 39 to be more suitable for *M. oryzae* conidia production than genotype Nipponbare. AUDS values were positively correlated to conidia production per lesion; however, the coefficient of determination was higher for the correlation with conidia production per lesion area. As the exponential equation gave the highest R² value, it may be difficult to exactly quantify very high sporulation rates of *M. oryzae* by hyperspectral measurements. However, as the linear relationship had almost the same R² value, the spectral difference quantified as AUDS seems to be suitable as a quantitative proxy for fungal spore production and enables an automated assessment of *M. oryzae* sporulation. Moreover, spectral analysis of fungal sporulation was also sensitive enough to characterize differences in the host quality of cultivars and in the aggressiveness of pathogen isolates.

Quantification of *M. oryzae* sporulation on leaf blast symptoms by hyperspectral imaging proved to be challenging, as HSI was not able to differentiate between hyaline mycelium, conidiophore, and conidia. Nonetheless, spectral differences were linked to variations in the number of *M. oryzae* conidia counted under the microscope. It is likely that a rather constant ratio between mycelial mass and number of conidia supports the quantitative assessment of *M. oryzae* sporulation. The coefficient of determination (R^2^ = 0.625) demonstrates the potential for improvement of the accuracy of automatic assessment of sporulation. Understanding and accounting for variations in the relation between mycelial mass and conidia production are essential for refining the quantification methods of fungal sporulation. The number of conidiophores per unit of area reflects the density of conidia produced by the pathogen, while the area of sporulation indicates the spatial extent of spore production on the leaf surface [20]. Incorporating information from both parameters, the AUDS approach allows for a more precise evaluation of the total number of conidia per blast lesion.

Hyperspectral imaging demonstrated its potential for the quantification of *M. oryzae* sporulation differing among rice genotypes. The high sensitivity of hyperspectral imaging enabled the detection of variation in spectral responses (of grey tissue) during sporulation depending on the rice × *M. oryzae* interaction. The integration of an HSI sensor with suitable data processing algorithms offers a more automated approach for the quantification of fungal sporulation. As conidia formation is a crucial step in blast epidemiology, the assessment of the partial resistance factor i.e., sporulation by hyperspectral techniques may improve the phenotyping of crops in breeding for disease resistance.

### Electronic supplementary material

Below is the link to the electronic supplementary material.

Supplementary Material 1

Supplementary Material 2

## Data Availability

All data generated or analysed during this study are included in this article [and its supplementary information files].
