# Supplementary material for: Hyperspectral imaging for quantifying Magnaporthe oryzae sporulation on rice genotypes
Source: Plant Methods. 2024 Jun 8;20:87. doi: 10.1186/s13007-024-01215-1 (PMC11161989; doi:10.1186/s13007-024-01215-1)
Supplement: Supplementary file 1 — Supplementary Material 1 [file 13007_2024_1215_MOESM1_ESM.docx]

**Supplementary Information**

**Additional file 1:**


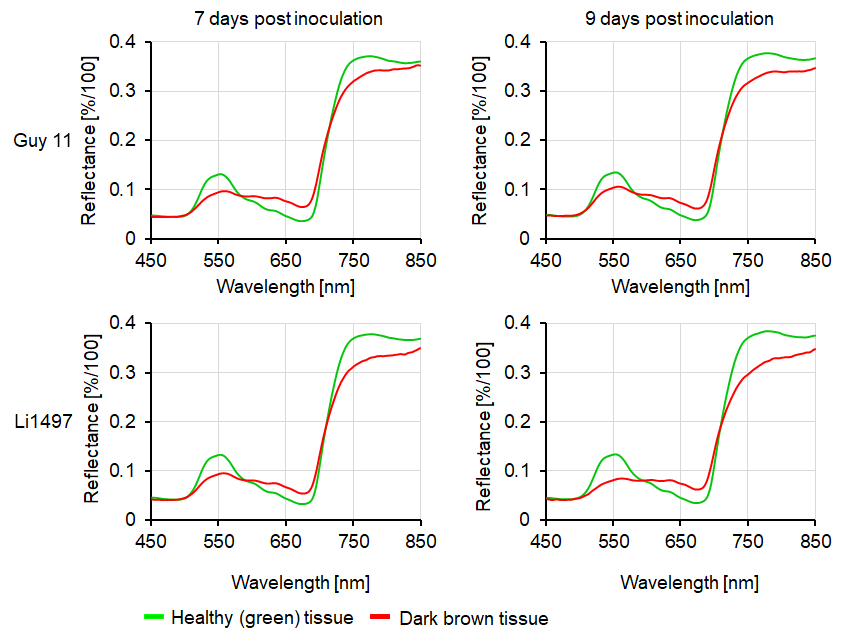


**Fig. S1:** Reference spectra of healthy (green) tissue and dark brown spots from leaves of rice genotype IR64 infected with *M. oryzae* isolates Guy 11 and Li1497, respectively. Spectra of green tissue and blast symptom type were extracted before (7 d.p.i.) and 2 days after induction of sporulation (= 9 d.p.i.).

**Additional file 2:**


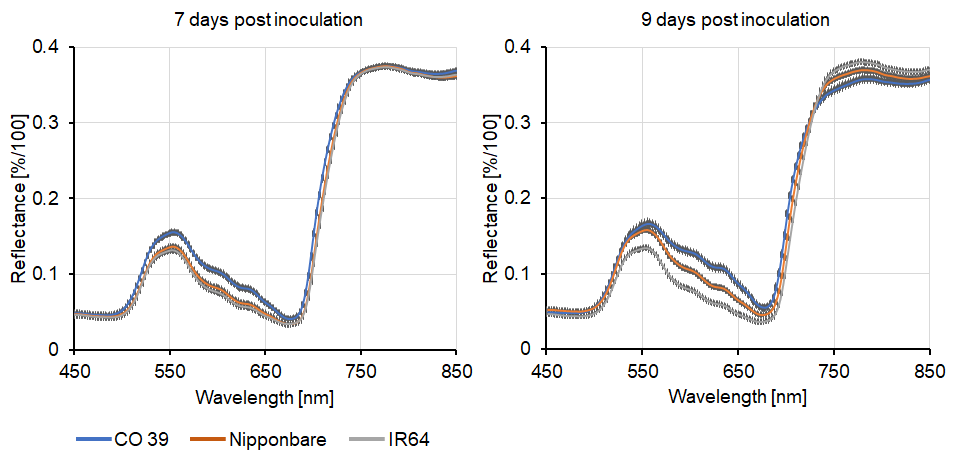


**Fig. S2:** Reflectance spectra of healthy (green) tissue of rice genotypes CO 39, Nipponbare and IR64 extracted before (7 d.p.i.) and 2 days after induction of sporulation (= 9 d.p.i.). For each waveband, the bars represent the standard error of the mean (n = 8).

**Additional file 3:**


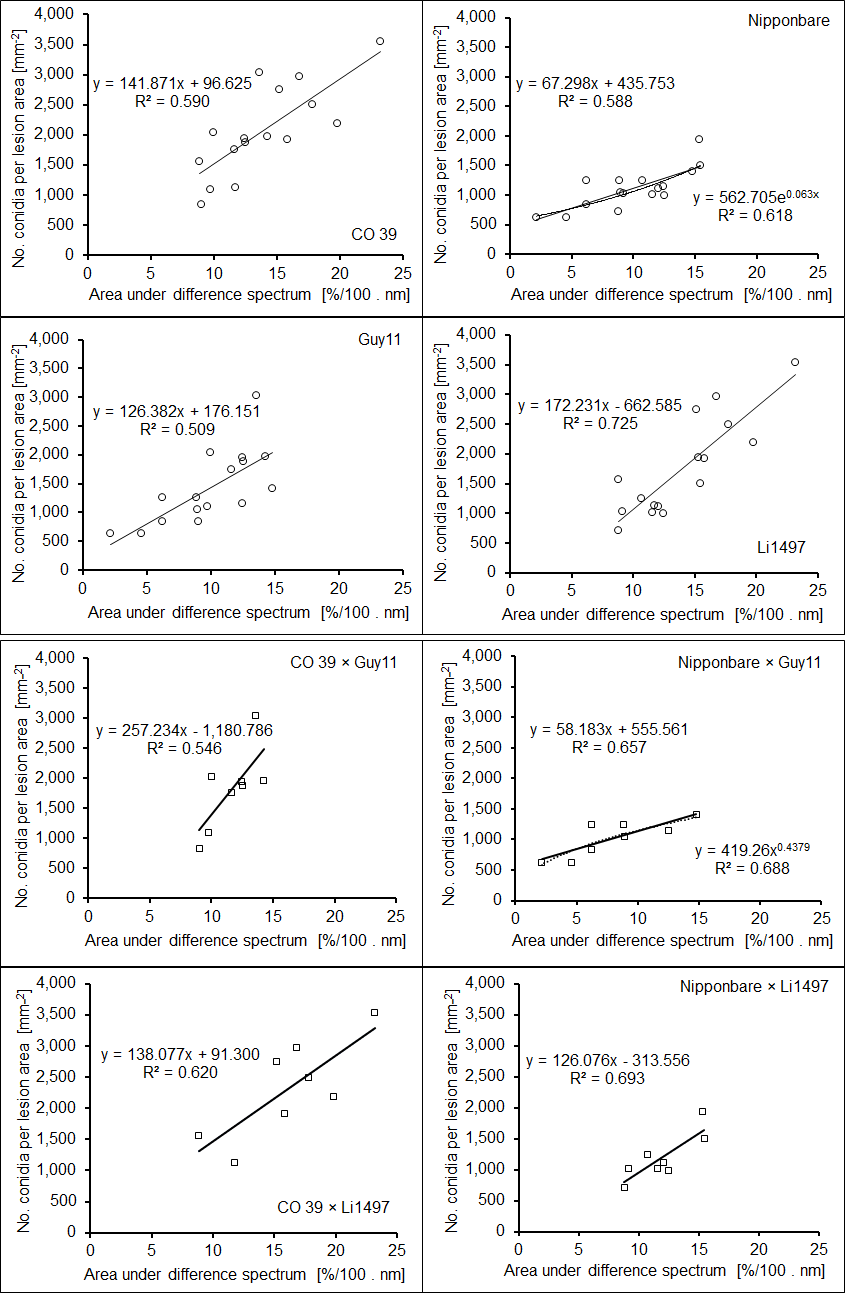


**Fig. S3:** Variability in the correlation between spectral data and conidia production by *M. oryzae* depending on the compatibility of the host-pathogen interaction (top two rows individual interactions; bottom two rows interactions depending on host genotype and pathogen genotype). Cv. CO 39 better supported sporulation than cv. Nipponbare; isolate Li1497 was more aggressive than isolate Guy 11.
