# Supplementary material for: Hyperspectral imaging for quantifying Magnaporthe oryzae sporulation on rice genotypes
Source: Plant Methods. 2024 Jun 8;20:87. doi: 10.1186/s13007-024-01215-1 (PMC11161989; doi:10.1186/s13007-024-01215-1)
Supplement: Supplementary file 2 — Supplementary Material 2 [file 13007_2024_1215_MOESM2_ESM.pptx]

## Slide 1
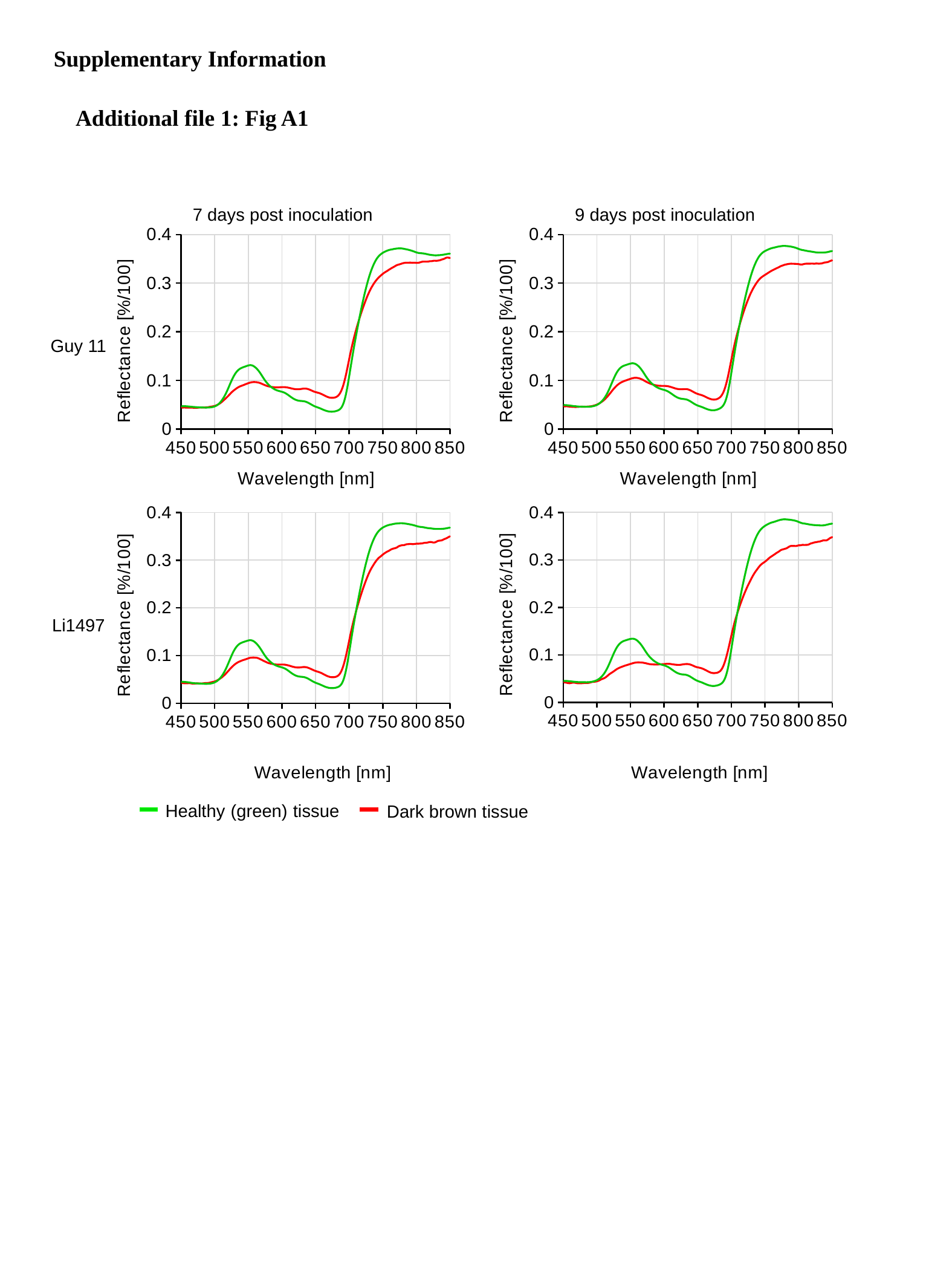

Supplementary Information
Additional file 1: Fig A1
7 days post inoculation
9 days post inoculation
### Chart
| Category | Healthy tissue | Dark brown |
|---|---|---|
### Chart
| Category | Healthy tissue | Dark brown |
|---|---|---|Guy 11
### Chart
| Category | Healthy tissue | Dark brown tissue |
|---|---|---|
### Chart
| Category | Healthy tissue | Dark brown tissue |
|---|---|---|Li1497
Healthy (green) tissue
Dark brown tissue

## Slide 2
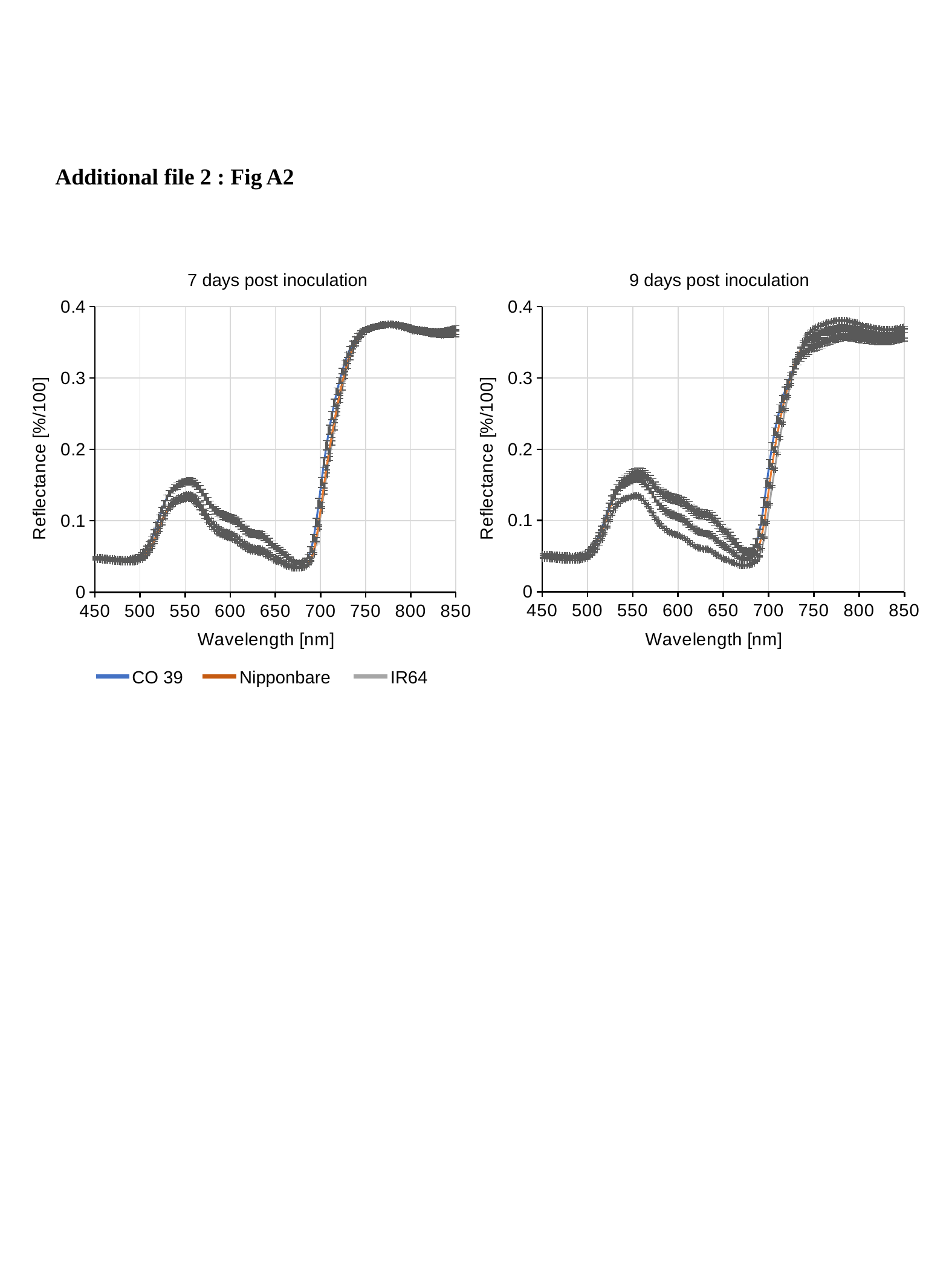

Additional file 2 : Fig A2
7 days post inoculation
9 days post inoculation
### Chart
| Category | CO 39 | Nipponbare | IR64 |
|---|---|---|---|
### Chart
| Category | CO 39 | Nipponbare | IR64 |
|---|---|---|---|CO 39
IR64
Nipponbare

## Slide 3
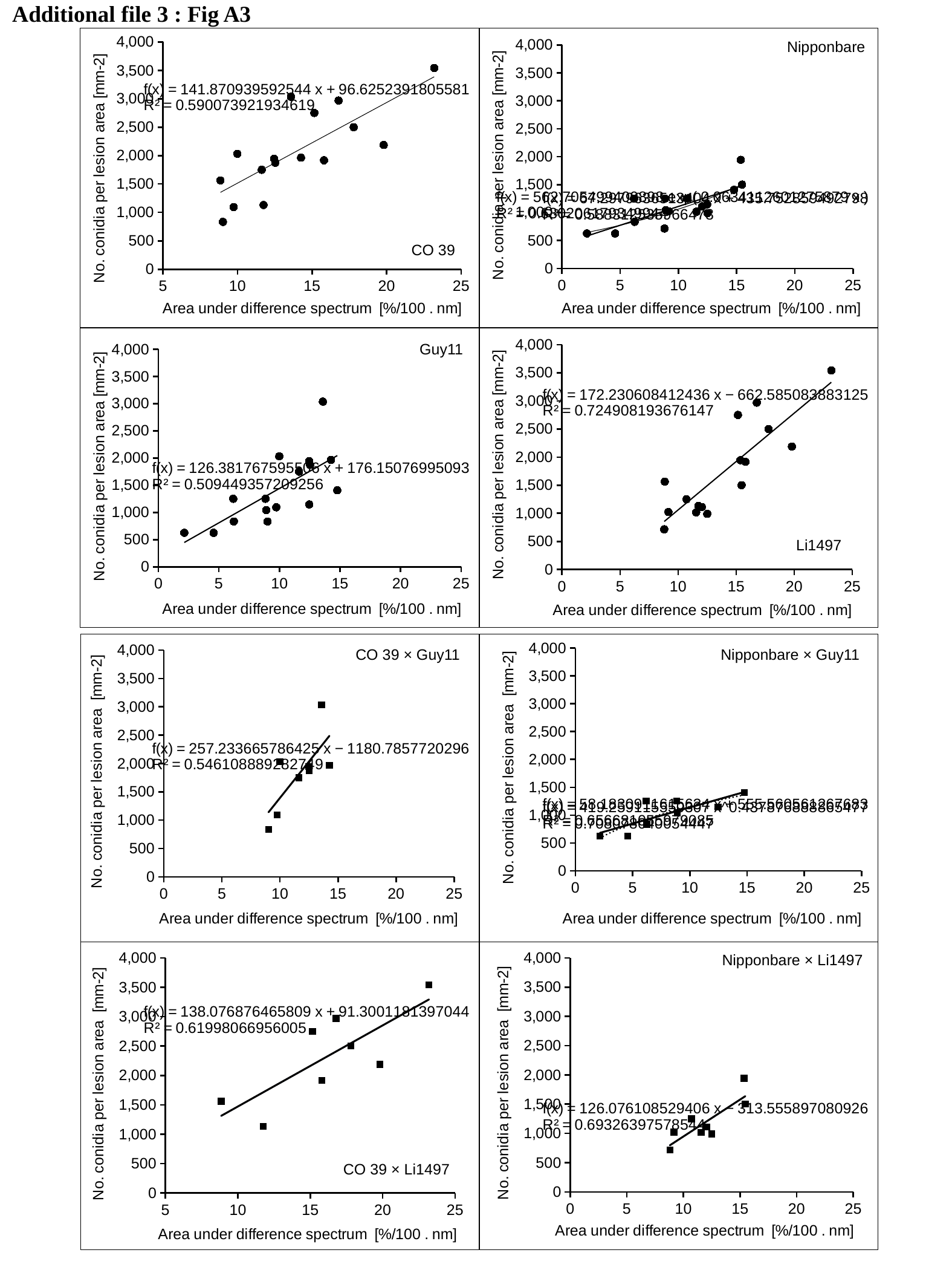

Additional file 3 : Fig A3
### Chart
| Category | |
|---|---|
### Chart
| Category | |
|---|---|Nipponbare
CO 39
### Chart
| Category | |
|---|---|
### Chart
| Category | |
|---|---|Guy11
Li1497
### Chart
| Category | No. of conidia per lesion area |
|---|---|
### Chart
| Category | No. of conidia per lesion area |
|---|---|CO 39 × Guy11
Nipponbare × Guy11
### Chart
| Category | No. of conidia per lesion area |
|---|---|
### Chart
| Category | No. of conidia per lesion area |
|---|---|Nipponbare × Li1497
CO 39 × Li1497
